# Supplementary material for: 3D variability analysis reveals a hidden conformational change controlling ammonia transport in human asparagine synthetase
Source: Nat Commun. 2024 Dec 3;15:10538. doi: 10.1038/s41467-024-54912-9 (PMC11615228; doi:10.1038/s41467-024-54912-9)
Supplement: Supplementary file 2 — Description of Additional Supplementary Files [file 41467_2024_54912_MOESM2_ESM.pdf]

Description of Additional Supplementary Files

File name: Supplementary Data 1
Description: Force field parameters for the b-aspartyl-AMP intermediate.

File name: Supplementary Data 2
Description: Script for analyzing and visualizing convergence of the well-tempered metadynamics simulations.

File name: Supplementary Data 3
Description: MD simulations checklist.
