# Supplementary material for: 3D variability analysis reveals a hidden conformational change controlling ammonia transport in human asparagine synthetase
Source: Nat Commun. 2024 Dec 3;15:10538. doi: 10.1038/s41467-024-54912-9 (PMC11615228; doi:10.1038/s41467-024-54912-9)
Supplement: Supplementary file 3 — Supplementary Data 1 [file 41467_2024_54912_MOESM3_ESM.pdf]

==
ATOMTYPES and PARAMETERS for molecule:
Structure4
==

Total charge of the system = -1.0000
Formal charge of the system = -1.0000

Charges

atom	formal	fractional formal
1	0	0.000
2	-1	-0.500
3	0	0.000
4	0	-0.500
5	0	0.000
6	0	0.000
7	0	0.000
8	0	0.000
9	0	0.000
10	0	0.000
11	0	0.000
12	0	0.000
13	0	0.000
14	0	0.000
15	0	0.000
16	0	0.000
17	0	0.000
18	0	0.000
19	0	0.000
20	0	0.000
21	0	0.000
22	0	0.000
23	0	0.000
24	0	0.000
25	0	0.000
26	0	0.000
27	0	0.000
28	0	0.000
29	1	1.000
30	0	-0.500
31	-1	-0.500
32	0	0.000
33	0	0.000
34	0	0.000
35	0	0.000
36	0	0.000
37	0	0.000
38	0	0.000
39	0	0.000
40	0	0.000
41	0	0.000
42	0	0.000
43	0	0.000
44	0	0.000
45	0	0.000
46	0	0.000
47	0	0.000
48	0	0.000
49	0	0.000

BCI's		index	type	type	bci	assignement info		
P1	O2	0	450	441	-0.4700	exact type	P2-O2Z	
P1	O3	0	450	452	-0.4100	exact type	P2-OS	
P1	O4	0	450	441	-0.4700	exact type	P2-O2Z	
P1	O5	0	450	452	-0.4100	exact type	P2-OS	
O3	C24	0	452	465	0.0800	score 180	OS-CO4	==> OS-C
O5	C6	0	452	135	0.2000	exact type	OS-CT	
C6	C7	0	135	181	0.0000	exact type	CT-CT	
C6	H32	0	135	140	0.0600	exact type	CT-HC	
C6	H33	0	135	140	0.0600	exact type	CT-HC	

C7	O8	0	181	180	-0.2000	exact type	CT-OS
C7	C9	0	181	135	-0.0000	exact type	CT-CT
C7	H34	0	181	140	0.0300	exact type	CT-HC
O8	C13	0	180	931	0.2000	exact type	OS-CO
C9	O10	0	135	154	-0.2650	exact type	CT-OH
C9	C11	0	135	135	0.0000	exact type	CT-CT
C9	H35	0	135	140	0.0600	exact type	CT-HC
O10	H36	0	154	240	0.4180	exact type	OH-HO
C11	O12	0	135	154	-0.2650	exact type	CT-OH
C11	C13	0	135	931	0.0000	empirical rule	CT-CO
C11	H37	0	135	140	0.0600	exact type	CT-HC
O12	H38	0	154	240	0.4180	exact type	OH-HO
C13	N14	0	931	354	-0.3500	exact type	CO-N*
C13	H39	0	931	140	0.1000	exact type	CO-HC
N14	C15	0	354	353	0.0796	exact type	N*-CRAB
N14	C23	0	354	349	0.0704	exact type	N*-C56A
C15	N16	0	353	352	-0.3204	exact type	CRAB-NNA
C15	H40	0	353	146	0.2000	exact type	CRAB-HA
N16	C17	0	352	350	0.1696	exact type	NNA-C56B
C17	C18	0	350	351	0.0333	exact type	C56B-CA
C17	C23	0	350	349	-0.0137	exact type	C56B-C56A
C18	N19	0	351	356	-0.0700	exact type	CA-NND
C18	N20	0	351	346	-0.3367	exact type	CA-NB
N19	H41	0	356	240	0.3700	exact type	NND-H
N19	H42	0	356	240	0.3700	exact type	NND-H
N20	C21	0	346	347	0.1933	exact type	NB-CQ
C21	N22	0	347	348	-0.2267	exact type	CQ-NB
C21	H43	0	347	146	0.2000	exact type	CQ-HA
N22	C23	0	348	349	0.3233	exact type	NB-C56A
C24	O25	0	465	281	-0.4300	exact type	CO4-O
C24	C26	0	465	135	-0.0000	exact type	CO4-CT
C26	C27	0	135	951	0.0000	empirical rule	CT-CT
C26	H44	0	135	140	0.0600	exact type	CT-HC
C26	H45	0	135	140	0.0600	exact type	CT-HC
C27	C28	0	951	952	0.0000	empirical rule	CT-CO3
C27	N29	0	951	286	-0.1225	empirical rule	CT-NP
C27	H46	0	951	140	0.0600	exact symbol	CT-HC
C28	O30	0	952	954	-0.2225	empirical rule	CO3-O2Z
C28	O31	0	952	954	-0.2225	empirical rule	CO3-O2Z
N29	H47	0	286	240	0.2100	empirical rule	NP-H
N29	H48	0	286	240	0.2100	empirical rule	NP-H
N29	H49	0	286	240	0.2100	empirical rule	NP-H

OPLSAA FORCE FIELD TYPE ASSIGNED

atom	type	vdw	symbol	charge	sigma	epsilon	quality	comment
P1	450	P1	P2	1.7600	3.7400	0.2000	high	P in PO2-
O2	441	O8	O2Z	-0.9700	2.9800	0.2000	high	O- in OP
O3	452	O16	OS	-0.4900	2.8500	0.1400	high	OR in PO3R-
O4	441	O8	O2Z	-0.9700	2.9800	0.2000	high	O= in OP
O5	452	O16	OS	-0.6100	2.8500	0.1400	high	OR in PO3R-
C6	135	C1	CT	0.0800	3.5000	0.0660	high	C: alkanes
C7	181	C1	CT	0.1700	3.5000	0.0660	high	C: ether
O8	180	O1	OS	-0.4000	2.9000	0.1400	high	O: ether
C9	135	C1	CT	0.2050	3.5000	0.0660	high	C: alkanes
O10	154	O3	OH	-0.6830	3.1200	0.1700	high	O: mono alcohols
C11	135	C1	CT	0.2050	3.5000	0.0660	high	C: alkanes
O12	154	O3	OH	-0.6830	3.1200	0.1700	high	O: mono alcohols
C13	931	C1	CO	0.4500	3.5000	0.0660	high	nucleosides
N14	354	N1	N*	-0.5000	3.2500	0.1700	high	Adenine N9
C15	353	C5	CRAB	0.2000	3.5000	0.0800	high	Adenine C8
N16	352	N1	NNA	-0.4900	3.2500	0.1700	high	Adenine N7
C17	350	C5	C56B	0.1500	3.5000	0.0800	high	Adenine C5
C18	351	C5	CA	0.4400	3.5000	0.0800	high	Adenine C6
N19	356	N1	NND	-0.8100	3.2500	0.1700	high	Adenine N-C6
N20	346	N1	NB	-0.5300	3.2500	0.1700	high	Adenine N1
C21	347	C5	CQ	0.2200	3.5000	0.0800	high	Adenine C2
N22	348	N1	NB	-0.5500	3.2500	0.1700	high	Adenine N3
C23	349	C5	C56A	0.3800	3.5000	0.0800	high	Adenine C4
C24	465	C2	CO4	0.5100	3.7500	0.1050	high	AA C: esters and thioesters

O25	281	O2	O	-0.4300	2.9600	0.2100	high	O: ketone, aldehyde, ester, acid
C26	135	C1	CT	-0.1200	3.5000	0.0660	high	C: alkanes
C27	951	C1	CT	0.0625	3.5000	0.0660	high	glycine zwit. 6-31G* CHELPG charges
C28	952	C2	CO3	0.4450	3.7500	0.1050	high	glycine zwit. 6-31G* CHELPG charges
N29	286	N7	NP	0.2475	3.2500	0.1700	high	glycine zwit. 6-31G* CHELPG charges
O30	954	O9	O2Z	-0.7225	3.1500	0.2500	high	glycine zwit. 6-31G* CHELPG charges
O31	954	O9	O2Z	-0.7225	3.1500	0.2500	high	glycine zwit. 6-31G* CHELPG charges
H32	140	H1	HC	0.0600	2.5000	0.0300	high	H: alkanes
H33	140	H1	HC	0.0600	2.5000	0.0300	high	H: alkanes
H34	140	H1	HC	0.0300	2.5000	0.0300	high	H: alkanes
H35	140	H1	HC	0.0600	2.5000	0.0300	high	H: alkanes
H36	240	H8	HO	0.4180	0.5000	0.0300	high	H(O): alcohols
H37	140	H1	HC	0.0600	2.5000	0.0300	high	H: alkanes
H38	240	H8	HO	0.4180	0.5000	0.0300	high	H(O): alcohols
H39	140	H4	HC	0.1000	2.5000	0.0500	high	Me A H-CN9
H40	146	H4	HA	0.2000	2.5000	0.0500	high	Adenine H-C8
H41	240	H8	H	0.3700	0.5000	0.0300	high	H on neutral N
H42	240	H8	H	0.3700	0.5000	0.0300	high	H on neutral N
H43	146	H4	HA	0.2000	2.5000	0.0500	high	Adenine H-C2
H44	140	H7	HC	0.0600	2.4200	0.0150	high	H on C-alpha in ether
H45	140	H7	HC	0.0600	2.4200	0.0150	high	H on C-alpha in ether
H46	140	H1	HC	0.0600	2.5000	0.0300	high	H: alkanes
H47	240	H8	H	0.2100	0.5000	0.0300	high	H attached to N+
H48	240	H8	H	0.2100	0.5000	0.0300	high	H attached to N+
H49	240	H8	H	0.2100	0.5000	0.0300	high	H attached to N+

Stretch		k	r0	quality	bt	comment	
P1	O2	525.00000	1.48000	high	0 => 0	P2-O2Z	==> P2-O2Z
P1	O3	230.00000	1.69700	high	0 => 0	P2-OS	==> P2-OS
P1	O4	525.00000	1.48000	high	0 => 0	P2-O2Z	==> P2-O2Z
P1	O5	230.00000	1.69700	high	0 => 0	P2-OS	==> P2-OS
O3	C24	214.00000	1.32700	high	0 => 0	OS-CO4	==> OS-CO4
O5	C6	320.00000	1.41000	high	0 => 0	OS-CT	==> OS-CT
C6	C7	268.00000	1.52900	high	0 => 0	CT-CT	==> CT-CT
C6	H32	340.00000	1.09000	high	0 => 0	CT-HC	==> HC-CT
C6	H33	340.00000	1.09000	high	0 => 0	CT-HC	==> HC-CT
C7	O8	320.00000	1.41000	high	0 => 0	CT-OS	==> OS-CT
C7	C9	268.00000	1.52900	high	0 => 0	CT-CT	==> CT-CT
C7	H34	340.00000	1.09000	high	0 => 0	CT-HC	==> HC-CT
O8	C13	320.00000	1.38000	high	0 => 0	OS-CO	==> OS-CO
C9	O10	320.00000	1.41000	high	0 => 0	CT-OH	==> OH-CT
C9	C11	268.00000	1.52900	high	0 => 0	CT-CT	==> CT-CT
C9	H35	340.00000	1.09000	high	0 => 0	CT-HC	==> HC-CT
O10	H36	553.00000	0.94500	high	0 => 0	OH-HO	==> OH-HO
C11	O12	320.00000	1.41000	high	0 => 0	CT-OH	==> OH-CT
C11	C13	250.00000	1.52700	high	0 => 0	CT-CO	==> CT-CO
C11	H37	340.00000	1.09000	high	0 => 0	CT-HC	==> HC-CT
O12	H38	553.00000	0.94500	high	0 => 0	OH-HO	==> OH-HO
C13	N14	275.00000	1.47900	high	0 => 0	CO-N*	==> N*-CO
C13	H39	340.00000	1.09000	high	0 => 0	CO-HC	==> HC-CO
N14	C15	440.00000	1.37100	high	0 => 0	N*-CRAB	==> N*-CRAB
N14	C23	436.00000	1.37400	high	0 => 0	N*-C56A	==> N*-C56A
C15	N16	529.00000	1.30400	high	0 => 0	CRAB-NNA	==> NNA-CRAB
C15	H40	367.00000	1.08000	high	0 => 0	CRAB-HA	==> HA-CRAB
N16	C17	414.00000	1.39100	high	0 => 0	NNA-C56B	==> NNA-C56B
C17	C18	469.00000	1.40400	high	0 => 0	C56B-CA	==> CA-C56B
C17	C23	447.00000	1.41900	high	0 => 0	C56B-C56A	==> C56B-C56A
C18	N19	481.00000	1.34000	high	0 => 0	CA-NND	==> NND-CA
C18	N20	483.00000	1.33900	high	0 => 0	CA-NB	==> NB-CA
N19	H41	434.00000	1.01000	high	0 => 0	NND-H	==> NND-H
N19	H42	434.00000	1.01000	high	0 => 0	NND-H	==> NND-H
N20	C21	502.00000	1.32400	high	0 => 0	NB-CQ	==> NB-CQ
C21	N22	502.00000	1.32400	high	0 => 0	CQ-NB	==> NB-CQ
C21	H43	367.00000	1.08000	high	0 => 0	CQ-HA	==> HA-CQ
N22	C23	461.00000	1.35400	high	0 => 0	NB-C56A	==> NB-C56A
C24	O25	570.00000	1.22900	high	0 => 0	CO4-O	==> O-CO4
C24	C26	317.00000	1.52200	high	0 => 0	CO4-CT	==> CT-CO4
C26	C27	268.00000	1.52900	high	0 => 0	CT-CT	==> CT-CT
C26	H44	340.00000	1.09000	high	0 => 0	CT-HC	==> HC-CT

C26	H45	340.00000	1.09000	high	0 => 0	CT-HC	==> HC-CT
C27	C28	317.00000	1.52200	high	0 => 0	CT-CO3	==> CT-CO3
C27	N29	367.00000	1.47100	high	0 => 0	CT-NP	==> NP-CT
C27	H46	340.00000	1.09000	high	0 => 0	CT-HC	==> HC-CT
C28	O30	656.00000	1.25000	high	0 => 0	CO3-O2Z	==> O2Z-CO3
C28	O31	656.00000	1.25000	high	0 => 0	CO3-O2Z	==> O2Z-CO3
N29	H47	434.00000	1.01000	high	0 => 0	NP-H	==> NP-H
N29	H48	434.00000	1.01000	high	0 => 0	NP-H	==> NP-H
N29	H49	434.00000	1.01000	high	0 => 0	NP-H	==> NP-H

Bending			k	theta0	quality	at	comment	
O2	P1	O3	100.00000	108.23000	high	0	O2Z-P2-OS	==> OS-P2-O2Z
O2	P1	O4	140.00000	119.90000	high	0	O2Z-P2-O2Z	==> O2Z-P2-O2Z
O2	P1	O5	100.00000	108.23000	high	0	O2Z-P2-OS	==> OS-P2-O2Z
O3	P1	O4	100.00000	108.23000	high	0	OS-P2-O2Z	==> OS-P2-O2Z
O3	P1	O5	45.00000	102.60000	high	0	OS-P2-OS	==> OS-P2-OS
O4	P1	O5	100.00000	108.23000	high	0	O2Z-P2-OS	==> OS-P2-O2Z
P1	O3	C24	80.00000	120.00000	med	0	P2-OS-CO4	==> C?-OS-P?
P1	O5	C6	100.00000	120.50000	high	0	P2-OS-CT	==> P2-OS-CT
O5	C6	C7	50.00000	109.50000	high	0	OS-CT-CT	==> OS-CT-CT
O5	C6	H32	35.00000	109.50000	high	0	OS-CT-HC	==> OS-CT-HC
O5	C6	H33	35.00000	109.50000	high	0	OS-CT-HC	==> OS-CT-HC
C7	C6	H32	37.50000	110.70000	high	0	CT-CT-HC	==> HC-CT-CT
C7	C6	H33	37.50000	110.70000	high	0	CT-CT-HC	==> HC-CT-CT
H32	C6	H33	33.00000	107.80000	high	0	HC-CT-HC	==> HC-CT-HC
C6	C7	O8	50.00000	109.50000	high	0	CT-CT-OS	==> OS-CT-CT
C6	C7	C9	58.35000	112.70000	high	0	CT-CT-CT	==> CT-CT-CT
C6	C7	H34	37.50000	110.70000	high	0	CT-CT-HC	==> HC-CT-CT
O8	C7	C9	50.00000	109.50000	high	0	OS-CT-CT	==> OS-CT-CT
O8	C7	H34	35.00000	109.50000	high	0	OS-CT-HC	==> OS-CT-HC
C9	C7	H34	37.50000	110.70000	high	0	CT-CT-HC	==> HC-CT-CT
C7	O8	C13	60.00000	109.50000	high	0	CT-OS-CO	==> CT-OS-CO
C7	C9	O10	50.00000	109.50000	high	0	CT-CT-OH	==> OH-CT-CT
C7	C9	C11	58.35000	112.70000	high	0	CT-CT-CT	==> CT-CT-CT
C7	C9	H35	37.50000	110.70000	high	0	CT-CT-HC	==> HC-CT-CT
O10	C9	C11	50.00000	109.50000	high	0	OH-CT-CT	==> OH-CT-CT
O10	C9	H35	35.00000	109.50000	high	0	OH-CT-HC	==> OH-CT-HC
C11	C9	H35	37.50000	110.70000	high	0	CT-CT-HC	==> HC-CT-CT
C9	O10	H36	55.00000	108.50000	high	0	CT-OH-HO	==> HO-OH-CT
C9	C11	O12	50.00000	109.50000	high	0	CT-CT-OH	==> OH-CT-CT
C9	C11	C13	75.00000	109.43000	high	0	CT-CT-CO	==> CT-CT-CO
C9	C11	H37	37.50000	110.70000	high	0	CT-CT-HC	==> HC-CT-CT
O12	C11	C13	90.00000	111.58700	high	0	OH-CT-CO	==> OH-CT-CO
O12	C11	H37	35.00000	109.50000	high	0	OH-CT-HC	==> OH-CT-HC
C13	C11	H37	45.00000	108.72000	high	0	CO-CT-HC	==> HC-CT-CO
C11	O12	H38	55.00000	108.50000	high	0	CT-OH-HO	==> HO-OH-CT
O8	C13	C11	90.00000	108.56700	high	0	OS-CO-CT	==> OS-CO-CT
O8	C13	N14	105.00000	106.22000	high	0	OS-CO-N*	==> OS-CO-N*
O8	C13	H39	35.00000	109.50000	high	0	OS-CO-HC	==> OS-CO-HC
C11	C13	N14	75.00000	107.44000	high	0	CT-CO-N*	==> N*-CO-CT
C11	C13	H39	45.00000	112.01100	high	0	CT-CO-HC	==> HC-CO-CT
N14	C13	H39	55.00000	103.44000	high	0	N*-CO-HC	==> N*-CO-HC
C13	N14	C15	70.00000	128.80000	high	0	CO-N*-CRAB	==> CRAB-N*-CT
C13	N14	C23	70.00000	125.80000	high	0	CO-N*-C56A	==> C56A-N*-CT
C15	N14	C23	70.00000	105.40000	high	0	CRAB-N*-C56A	==> CRAB-N*-C56A
N14	C15	N16	70.00000	113.90000	high	0	N*-CRAB-NNA	==> NNA-CRAB-N*
N14	C15	H40	35.00000	123.05000	high	0	N*-CRAB-HA	==> N*-CRAB-HA
N16	C15	H40	35.00000	123.05000	high	0	NNA-CRAB-HA	==> NNA-CRAB-HA
C15	N16	C17	70.00000	103.80000	high	0	CRAB-NNA-C56B	==> CRAB-NNA-C56B
N16	C17	C18	70.00000	132.40000	high	0	NNA-C56B-CA	==> NNA-C56B-CA
N16	C17	C23	70.00000	110.40000	high	0	NNA-C56B-C56A	==> NNA-C56B-C56A
C18	C17	C23	85.00000	116.20000	high	0	CA-C56B-C56A	==> CA-C56B-C56A
C17	C18	N19	70.00000	123.50000	high	0	C56B-CA-NND	==> NND-CA-C56B
C17	C18	N20	70.00000	117.30000	high	0	C56B-CA-NB	==> NB-CA-C56B
N19	C18	N20	70.00000	119.30000	high	0	NND-CA-NB	==> NND-CA-NB
C18	N19	H41	35.00000	120.00000	high	0	CA-NND-H	==> H-NND-CA
C18	N19	H42	35.00000	120.00000	high	0	CA-NND-H	==> H-NND-CA
H41	N19	H42	35.00000	120.00000	high	0	H-NND-H	==> H-NND-H
C18	N20	C21	70.00000	118.60000	high	0	CA-NB-CQ	==> CQ-NB-CA

N20	C21	N22	70.00000	129.10000	high	0	NB-CQ-NB	==> NB-CQ-NB
N20	C21	H43	35.00000	115.45000	high	0	NB-CQ-HA	==> NB-CQ-HA
N22	C21	H43	35.00000	115.45000	high	0	NB-CQ-HA	==> NB-CQ-HA
C21	N22	C23	70.00000	111.00000	high	0	CQ-NB-C56A	==> CQ-NB-C56A
N14	C23	C17	70.00000	106.20000	high	0	N*-C56A-C56B	==> N*-C56A-C56B
N14	C23	N22	70.00000	126.20000	high	0	N*-C56A-NB	==> NB-C56A-N*
C17	C23	N22	70.00000	127.70000	high	0	C56B-C56A-NB	==> NB-C56A-C56B
O3	C24	O25	83.00000	123.40000	high	0	OS-CO4-O	==> OS-CO4-O
O3	C24	C26	81.00000	111.40000	high	0	OS-CO4-CT	==> OS-CO4-CT
O25	C24	C26	80.00000	120.40000	high	0	O-CO4-CT	==> O-CO4-CT
C24	C26	C27	63.00000	111.10000	high	0	CO4-CT-CT	==> CT-CT-CO4
C24	C26	H44	35.00000	109.50000	high	0	CO4-CT-HC	==> HC-CT-CO4
C24	C26	H45	35.00000	109.50000	high	0	CO4-CT-HC	==> HC-CT-CO4
C27	C26	H44	37.50000	110.70000	high	0	CT-CT-HC	==> HC-CT-CT
C27	C26	H45	37.50000	110.70000	high	0	CT-CT-HC	==> HC-CT-CT
H44	C26	H45	33.00000	107.80000	high	0	HC-CT-HC	==> HC-CT-HC
C26	C27	C28	70.00000	109.69300	high	0	CT-CT-CO3	==> CT-CT-CO3
C26	C27	N29	80.00000	111.20000	high	0	CT-CT-NP	==> NP-CT-CT
C26	C27	H46	37.50000	110.70000	high	0	CT-CT-HC	==> HC-CT-CT
C28	C27	N29	80.00000	109.26000	high	0	CO3-CT-NP	==> C-CT-NP
C28	C27	H46	35.00000	109.50000	high	0	CO3-CT-HC	==> HC-CT-CO3
N29	C27	H46	35.00000	109.50000	high	0	NP-CT-HC	==> NP-CT-HC
C27	C28	O30	70.00000	117.00000	high	0	CT-CO3-O2Z	==> O2Z-CO3-CT
C27	C28	O31	70.00000	117.00000	high	0	CT-CO3-O2Z	==> O2Z-CO3-CT
O30	C28	O31	80.00000	126.00000	high	0	O2Z-CO3-O2Z	==> O2Z-CO3-O2Z
C27	N29	H47	35.00000	109.50000	high	0	CT-NP-H	==> H-NP-CT
C27	N29	H48	35.00000	109.50000	high	0	CT-NP-H	==> H-NP-CT
C27	N29	H49	35.00000	109.50000	high	0	CT-NP-H	==> H-NP-CT
H47	N29	H48	35.00000	109.50000	high	0	H-NP-H	==> H-NP-H
H47	N29	H49	35.00000	109.50000	high	0	H-NP-H	==> H-NP-H
H48	N29	H49	35.00000	109.50000	high	0	H-NP-H	==> H-NP-H

proper Torsion				V1	V2	V3	V4	quality	tt	comment
O2	P1	O3	C24	0.000	0.000	0.000	0.000	med	0	O2Z-P2-OS-CO4 ==> O2Z-P2-OS-??
O4	P1	O3	C24	0.000	0.000	0.000	0.000	med	0	O2Z-P2-OS-CO4 ==> O2Z-P2-OS-??
O5	P1	O3	C24	0.000	0.000	0.300	0.000	low	0	OS-P2-OS-CO4 ==> ??-OS-P2-??
O2	P1	O5	C6	0.000	0.000	0.000	0.000	high	0	O2Z-P2-OS-CT ==> O2Z-P2-OS-CT
O3	P1	O5	C6	0.101	-2.614	1.831	0.000	high	0	OS-P2-OS-CT ==> OS-P2-OS-CT
O4	P1	O5	C6	0.000	0.000	0.000	0.000	high	0	O2Z-P2-OS-CT ==> O2Z-P2-OS-CT
P1	O3	C24	O25	0.000	6.000	0.000	0.000	high	0	P2-OS-CO4-O ==> ??-OS-C-O
P1	O3	C24	C26	0.000	4.000	0.000	0.000	med	0	P2-OS-CO4-CT ==> ??-OS-C-??
P1	O5	C6	C7	-5.311	-0.447	0.483	0.000	high	0	P2-OS-CT-CT ==> P2-OS-CT-CT
P1	O5	C6	H32	0.000	0.000	0.060	0.000	high	0	P2-OS-CT-HC ==> P?-OS-CT-HC
P1	O5	C6	H33	0.000	0.000	0.060	0.000	high	0	P2-OS-CT-HC ==> P?-OS-CT-HC
O5	C6	C7	O8	2.238	-2.327	-0.683	0.000	high	0	OS-CT-CT-OS ==> OS-CT-CT-OS
O5	C6	C7	C9	1.255	-0.619	0.546	0.000	high	0	OS-CT-CT-CT ==> OS-CT-CT-CT
O5	C6	C7	H34	0.000	0.000	0.468	0.000	high	0	OS-CT-CT-HC ==> O?-CT-CT-HC
H32	C6	C7	O8	0.000	0.000	0.468	0.000	high	0	HC-CT-CT-OS ==> O?-CT-CT-HC
H32	C6	C7	C9	0.000	0.000	0.300	0.000	high	0	HC-CT-CT-CT ==> HC-CT-CT-CT
H32	C6	C7	H34	0.000	0.000	0.300	0.000	high	0	HC-CT-CT-HC ==> HC-CT-CT-HC
H33	C6	C7	O8	0.000	0.000	0.468	0.000	high	0	HC-CT-CT-OS ==> O?-CT-CT-HC
H33	C6	C7	C9	0.000	0.000	0.300	0.000	high	0	HC-CT-CT-CT ==> HC-CT-CT-CT

```
 H33     C6      C7      H34       0.000   0.000   0.300   0.000    high   0   HC-CT-CT-HC
==> HC-CT-CT-HC
 C6      C7      O8      C13       0.125  -0.022   0.561   0.000    high   0   CT-CT-OS-CO
==> CT-OS-CT-CT
 C9      C7      O8      C13       0.125  -0.022   0.561   0.000    high   0   CT-CT-OS-CO
==> CT-OS-CT-CT
 H34     C7      O8      C13       0.000   0.000   0.741   0.000    high   0   HC-CT-OS-CO
==> CT-OS-CT-HC
 C6      C7      C9      O10       1.730  -0.350   0.416   0.000    high   0   CT-CT-CT-OH
==> OH-CT-CT-CT
 C6      C7      C9      C11       1.135  -0.151   0.400   0.000    high   0   CT-CT-CT-CT
==> CT-CT-CT-CT
 C6      C7      C9      H35       0.000   0.000   0.300   0.000    high   0   CT-CT-CT-HC
==> HC-CT-CT-CT
 O8      C7      C9      O10       7.387  -3.659   0.000   0.000    high   0   OS-CT-CT-OH
==> OS-CT-CT-OH
 O8      C7      C9      C11       1.255  -0.619   0.546   0.000    high   0   OS-CT-CT-CT
==> OS-CT-CT-CT
 O8      C7      C9      H35       0.000   0.000   0.468   0.000    high   0   OS-CT-CT-HC
==> O?-CT-CT-HC
 H34     C7      C9      O10       0.000   0.000   0.468   0.000    high   0   HC-CT-CT-OH
==> O?-CT-CT-HC
 H34     C7      C9      C11       0.000   0.000   0.300   0.000    high   0   HC-CT-CT-CT
==> HC-CT-CT-CT
 H34     C7      C9      H35       0.000   0.000   0.300   0.000    high   0   HC-CT-CT-HC
==> HC-CT-CT-HC
 C7      O8      C13     C11       0.125  -0.022   0.561   0.000    high   0   CT-OS-CO-CT
==> CT-OS-CT-CT
 C7      O8      C13     N14      -4.755  -0.163   1.180   0.000    high   0   CT-OS-CO-N*
==> CT-OS-CT-N
 C7      O8      C13     H39       0.000   0.000   0.741   0.000    high   0   CT-OS-CO-HC
==> CT-OS-CT-HC
 C7      C9      O10     H36      -0.232  -0.189   0.425   0.000    high   0   CT-CT-OH-HO
==> HO-OH-CT-CT
 C11     C9      O10     H36      -0.232  -0.189   0.425   0.000    high   0   CT-CT-OH-HO
==> HO-OH-CT-CT
 H35     C9      O10     H36       0.000   0.000   0.389   0.000    high   0   HC-CT-OH-HO
==> HO-OH-CT-HC
 C7      C9      C11     O12       1.730  -0.350   0.416   0.000    high   0   CT-CT-CT-OH
==> OH-CT-CT-CT
 C7      C9      C11     C13       7.941  -0.000   0.000   0.000    high   0   CT-CT-CT-CO
==> CT-CT-CT-CO
 C7      C9      C11     H37       0.000   0.000   0.300   0.000    high   0   CT-CT-CT-HC
==> HC-CT-CT-CT
 O10     C9      C11     O12       7.509   0.000   0.000   0.000    high   0   OH-CT-CT-OH
==> OH-CT-CT-OH
 O10     C9      C11     C13       1.730  -0.350   0.416   0.000    high   0   OH-CT-CT-CO
==> OH-CT-CT-CT
 O10     C9      C11     H37       0.000   0.000   0.468   0.000    high   0   OH-CT-CT-HC
==> O?-CT-CT-HC
 H35     C9      C11     O12       0.000   0.000   0.468   0.000    high   0   HC-CT-CT-OH
==> O?-CT-CT-HC
 H35     C9      C11     C13       0.000   0.000   0.300   0.000    high   0   HC-CT-CT-CO
==> HC-CT-CT-CT
 H35     C9      C11     H37       0.000   0.000   0.300   0.000    high   0   HC-CT-CT-HC
==> HC-CT-CT-HC
 C9      C11     O12     H38      -0.232  -0.189   0.425   0.000    high   0   CT-CT-OH-HO
==> HO-OH-CT-CT
 C13     C11     O12     H38      -0.232  -0.189   0.425   0.000    high   0   CO-CT-OH-HO
==> HO-OH-CT-CT
 H37     C11     O12     H38       0.000   0.000   0.389   0.000    high   0   HC-CT-OH-HO
==> HO-OH-CT-HC
 C9      C11     C13     O8        1.255  -0.619   0.546   0.000    high   0   CT-CT-CO-OS
==> OS-CT-CT-CT
 C9      C11     C13     N14      -1.292   1.168   0.000   0.000    high   0   CT-CT-CO-N*
==> NA-CT-CT-CT
 C9      C11     C13     H39       0.000   0.000   0.300   0.000    high   0   CT-CT-CO-HC
==> HC-CT-CT-CT
 O12     C11     C13     O8        7.387  -3.659   0.000   0.000    high   0   OH-CT-CO-OS
==> OS-CT-CT-OH
```

```
 O12     C11     C13     N14       5.538  -0.487  -1.565   0.000    high   0    OH-CT-CO-N*
==> OH-CT-CT-N
 O12     C11     C13     H39       0.000   0.000   0.468   0.000    high   0    OH-CT-CO-HC
==> O?-CT-CT-HC
 H37     C11     C13     O8        0.000   0.000   0.468   0.000    high   0    HC-CT-CO-OS
==> O?-CT-CT-HC
 H37     C11     C13     N14       0.000   0.000   0.300   0.000    high   0    HC-CT-CO-N*
==> HC-CT-CT-??
 H37     C11     C13     H39       0.000   0.000   0.300   0.000    high   0    HC-CT-CO-HC
==> HC-CT-CT-HC
 O8      C13     N14     C15       0.207  -0.307   0.000   0.000    high   0    OS-CO-N*-CRAB
==> C?-NA-CT-OS
 O8      C13     N14     C23       0.207  -0.307   0.000   0.000    high   0    OS-CO-N*-C56A
==> C?-NA-CT-OS
 C11     C13     N14     C15       2.205   0.033   0.782   0.000    high   0    CT-CO-N*-CRAB
==> CA-NA-CT-CT
 C11     C13     N14     C23       2.205   0.033   0.782   0.000    high   0    CT-CO-N*-C56A
==> CA-NA-CT-CT
 H39     C13     N14     C15       0.000   0.000   0.000   0.000    high   0    HC-CO-N*-CRAB
==> ??-NA-CT-HC
 H39     C13     N14     C23       0.000   0.000   0.000   0.000    high   0    HC-CO-N*-C56A
==> ??-NA-CT-HC
 C13     N14     C15     N16       0.000   7.250   0.000   0.000    high   0    CO-N*-CRAB-NNA
==> ??-NA-CA-??
 C13     N14     C15     H40       0.000   7.250   0.000   0.000    high   0    CO-N*-CRAB-HA
==> ??-NA-CA-??
 C23     N14     C15     N16       0.000   7.250   0.000   0.000    high   0    C56A-N*-CRAB-NNA
==> ??-NA-CA-??
 C23     N14     C15     H40       0.000   7.250   0.000   0.000    high   0    C56A-N*-CRAB-HA
==> ??-NA-CA-??
 C13     N14     C23     C17       1.628   3.111   0.000   0.000    high   0    CO-N*-C56A-C56B
==> CT-N-CA-CA
 C13     N14     C23     N22       0.000   7.250   0.000   0.000    high   0    CO-N*-C56A-NB
==> ??-NA-CA-??
 C15     N14     C23     C17       1.405   3.123   0.000   0.000    med    0    CRAB-N*-C56A-C56B
==> C?-N-CA-CA
 C15     N14     C23     N22       0.000   7.250   0.000   0.000    high   0    CRAB-N*-C56A-NB
==> ??-NA-CA-??
 N14     C15     N16     C17       0.000   7.250   0.000   0.000    high   0    N*-CRAB-NNA-C56B
==> CA-NNA-CA-NB
 H40     C15     N16     C17       0.000   7.250   0.000   0.000    high   0    HA-CRAB-NNA-C56B
==> CA-NNA-CA-HA
 C15     N16     C17     C18       0.000   7.250   0.000   0.000    high   0    CRAB-NNA-C56B-CA
==> CA-NNA-CA-CA
 C15     N16     C17     C23       0.000   7.250   0.000   0.000    high   0    CRAB-NNA-C56B-
C56A   ==> CA-NNA-CA-CA
 N16     C17     C18     N19       0.000   7.250   0.000   0.000    high   0    NNA-C56B-CA-NND
==> ??-CA-CA-??
 N16     C17     C18     N20       0.000   7.250   0.000   0.000    high   0    NNA-C56B-CA-NB
==> ??-CA-CA-??
 C23     C17     C18     N19       0.000   7.250   0.000   0.000    high   0    C56A-C56B-CA-NND
==> ??-CA-CA-??
 C23     C17     C18     N20       0.000   7.250   0.000   0.000    high   0    C56A-C56B-CA-NB
==> ??-CA-CA-??
 N16     C17     C23     N14       0.000   7.250   0.000   0.000    high   0    NNA-C56B-C56A-N*
==> ??-CA-CA-??
 N16     C17     C23     N22       0.000   7.250   0.000   0.000    high   0    NNA-C56B-C56A-NB
==> ??-CA-CA-??
 C18     C17     C23     N14       0.000   6.000   0.000   0.000    high   0    CA-C56B-C56A-N*
==> CA-C56B-C56A-NA
 C18     C17     C23     N22       0.000   7.250   0.000   0.000    high   0    CA-C56B-C56A-NB
==> ??-CA-CA-??
 C17     C18     N19     H41       0.000   1.400   0.000   0.000    high   0    C56B-CA-NND-H
==> H-NND-CA-CA
 C17     C18     N19     H42       0.000   1.400   0.000   0.000    high   0    C56B-CA-NND-H
==> H-NND-CA-CA
 N20     C18     N19     H41       0.000   1.400   0.000   0.000    high   0    NB-CA-NND-H
==> H-NND-CA-NB
 N20     C18     N19     H42       0.000   1.400   0.000   0.000    high   0    NB-CA-NND-H
==> H-NND-CA-NB
```

```
 C17     C18     N20     C21       0.000   7.250   0.000   0.000    high   0    C56B-CA-NB-CQ
==> ??-NB-CA-??
 N19     C18     N20     C21       0.000   7.250   0.000   0.000    high   0    NND-CA-NB-CQ
==> ??-NB-CA-??
 C18     N20     C21     N22       0.000   7.250   0.000   0.000    high   0    CA-NB-CQ-NB
==> ??-NB-CA-??
 C18     N20     C21     H43       0.000   7.250   0.000   0.000    high   0    CA-NB-CQ-HA
==> ??-NB-CA-??
 N20     C21     N22     C23       0.000   7.250   0.000   0.000    high   0    NB-CQ-NB-C56A
==> ??-NB-CA-??
 H43     C21     N22     C23       0.000   7.250   0.000   0.000    high   0    HA-CQ-NB-C56A
==> ??-NB-CA-??
 C21     N22     C23     N14       0.000   7.250   0.000   0.000    high   0    CQ-NB-C56A-N*
==> ??-NB-CA-??
 C21     N22     C23     C17       0.000   7.250   0.000   0.000    high   0    CQ-NB-C56A-C56B
==> ??-NB-CA-??
 O3      C24     C26     C27       0.000   0.000   0.735   0.000    high   0    OS-CO4-CT-CT
==> CT-CT-C-OS
 O3      C24     C26     H44       0.000   0.000   0.000   0.000    high   0    OS-CO4-CT-HC
==> HC-CT-C-??
 O3      C24     C26     H45       0.000   0.000   0.000   0.000    high   0    OS-CO4-CT-HC
==> HC-CT-C-??
 O25     C24     C26     C27       0.000   0.603   0.000   0.000    high   0    O-CO4-CT-CT
==> ??-CT-C-O
 O25     C24     C26     H44       0.000   0.000   0.000   0.000    high   0    O-CO4-CT-HC
==> HC-CT-C-O
 O25     C24     C26     H45       0.000   0.000   0.000   0.000    high   0    O-CO4-CT-HC
==> HC-CT-C-O
 C24     C26     C27     C28      -0.619   2.223   0.807   0.000    high   0    CO4-CT-CT-CO3
==> CO3-CT-CT-C
 C24     C26     C27     N29      -2.931  -0.612  -0.119   0.000    high   0    CO4-CT-CT-NP
==> NP-CT-CT-C
 C24     C26     C27     H46       0.000   0.000  -0.225   0.000    high   0    CO4-CT-CT-HC
==> HC-CT-CT-C
 H44     C26     C27     C28       0.000   0.000  -0.225   0.000    high   0    HC-CT-CT-CO3
==> HC-CT-CT-C
 H44     C26     C27     N29       0.000   0.000   0.384   0.000    high   0    HC-CT-CT-NP
==> NP-CT-CT-HC
 H44     C26     C27     H46       0.000   0.000   0.300   0.000    high   0    HC-CT-CT-HC
==> HC-CT-CT-HC
 H45     C26     C27     C28       0.000   0.000  -0.225   0.000    high   0    HC-CT-CT-CO3
==> HC-CT-CT-C
 H45     C26     C27     N29       0.000   0.000   0.384   0.000    high   0    HC-CT-CT-NP
==> NP-CT-CT-HC
 H45     C26     C27     H46       0.000   0.000   0.300   0.000    high   0    HC-CT-CT-HC
==> HC-CT-CT-HC
 C26     C27     C28     O30       0.000   0.823   0.000   0.000    high   0    CT-CT-CO3-O2Z
==> CT-CT-CO3-O2Z
 C26     C27     C28     O31       0.000   0.823   0.000   0.000    high   0    CT-CT-CO3-O2Z
==> CT-CT-CO3-O2Z
 N29     C27     C28     O30       0.000   0.603   0.000   0.000    high   0    NP-CT-CO3-O2Z
==> ??-CT-C-O
 N29     C27     C28     O31       0.000   0.603   0.000   0.000    high   0    NP-CT-CO3-O2Z
==> ??-CT-C-O
 H46     C27     C28     O30       0.000   0.000   0.000   0.000    high   0    HC-CT-CO3-O2Z
==> HC-CT-CO3-O2Z
 H46     C27     C28     O31       0.000   0.000   0.000   0.000    high   0    HC-CT-CO3-O2Z
==> HC-CT-CO3-O2Z
 C26     C27     N29     H47       0.000   0.000   0.347   0.000    high   0    CT-CT-NP-H
==> H-NP-CT-CT
 C26     C27     N29     H48       0.000   0.000   0.347   0.000    high   0    CT-CT-NP-H
==> H-NP-CT-CT
 C26     C27     N29     H49       0.000   0.000   0.347   0.000    high   0    CT-CT-NP-H
==> H-NP-CT-CT
 C28     C27     N29     H47       0.000   0.000   0.347   0.000    high   0    CO3-CT-NP-H
==> H-NP-CT-??
 C28     C27     N29     H48       0.000   0.000   0.347   0.000    high   0    CO3-CT-NP-H
==> H-NP-CT-??
 C28     C27     N29     H49       0.000   0.000   0.347   0.000    high   0    CO3-CT-NP-H
==> H-NP-CT-??
```

H46	C27	N29	H47	0.000	0.000	0.261	0.000	high	0	HC-CT-NP-H ==> H-NP-CT-HC
H46	C27	N29	H48	0.000	0.000	0.261	0.000	high	0	HC-CT-NP-H ==> H-NP-CT-HC
H46	C27	N29	H49	0.000	0.000	0.261	0.000	high	0	HC-CT-NP-H ==> H-NP-CT-HC

improper Torsion				V2	quality	comment
C15	C23	N14	C13	8.000	high	aromatic atom
N16	H40	C15	N14	2.200	high	aromatic atom
C18	C23	C17	N16	8.000	high	aromatic atom
N19	N20	C18	C17	8.000	high	aromatic atom
H42	C18	N19	H41	2.000	high	amino substituted aromatic rings
N22	H43	C21	N20	2.200	high	aromatic atom
C17	N22	C23	N14	8.000	high	aromatic atom
O3	C26	C24	O25	21.000	high	carbonyl and analogs
O31	C27	C28	O30	21.000	high	carboxylate
