# Supplementary material for: 3D variability analysis reveals a hidden conformational change controlling ammonia transport in human asparagine synthetase
Source: Nat Commun. 2024 Dec 3;15:10538. doi: 10.1038/s41467-024-54912-9 (PMC11615228; doi:10.1038/s41467-024-54912-9)
Supplement: Supplementary file 4 — Supplementary Data 2 [file 41467_2024_54912_MOESM4_ESM.pdf]

```
"""
Script to analyze a metadynamics simulation and determine if it has
converged.

The script will generate a PDF report and will including a number of plots
including:

* The FES as a function of time
* Overlay in the common region
* Free energy difference across different FES evaluations
* Average FES
* Standard deviation of the FES
* Average difference between different FES snapshots
* Distribution of standard deviations

Copyright Schrodinger, LLC. All rights reserved.
"""
from __future__ import annotations

import sys
import copy
import math

import numpy
import argparse
import reportlab
import statistics

from collections import defaultdict

from reportlab.lib import units
from reportlab.lib import enums
from reportlab.lib import styles

from reportlab import platypus

from schrodinger.utils import sea
from schrodinger.utils import cmdline
from schrodinger.utils import fileutils

from schrodinger.application.desmond import meta
from schrodinger.application.desmond import mplchart
from schrodinger.application.desmond import constants

from schrodinger.application.matsci import parserutils

from  pprint import pprint

my_style = styles.getSampleStyleSheet()

TABLE_STYLE = platypus.TableStyle([('VALIGN', (0, 0), (0, 0), 'TOP'),
                                   ('VALIGN', (1, 0), (1, 0), 'MIDDLE')])

PARA_STYLE = styles.ParagraphStyle('Normal',
                                   alignment=enums.TA_JUSTIFY,
                                   fontSize=8)
```

```
SQUARE_IMG = (2 * units.inch, 2 * units.inch)
RECTANGLE_IMG = (3.45 * units.inch, 2.3 * units.inch)


class PlotInfo:
    """
    Plot information
    """
    height_header = platypus.Paragraph('Hills Height as a Function of Time',
                                       my_style['Heading2'])

    time_header = platypus.Paragraph('Free Energy as a Function of Time',
                                     my_style['Heading2'])

    overlay_header = platypus.Paragraph('Free Energy Overlays',
                                        my_style['Heading2'])

    avg_header = platypus.Paragraph('Average Free Energy Surface',
                                    my_style['Heading2'])

    err_with_time_header = platypus.Paragraph(
        'Distance of Free Energies as a Function of Time',
my_style['Heading2'])

    prob_err_with_time_header = platypus.Paragraph(
        'Distance of Probability Density per Block as a Function of Time',
        my_style['Heading2'])

    stdev_1D_header = platypus.Paragraph(
        'Distribution of Standard Deviations Along the Surface',
        my_style['Heading2'])

    stdev_2D_header = platypus.Paragraph('Standard Deviations Surface',
                                         my_style['Heading2'])

    block_probabilities_header = platypus.Paragraph('Block Probabilities',
                                                    my_style['Heading2'])

    desc_height = 'In Well-Tempered Metadynamics, it\'s important that the '
\
                  'hills decay to a very low value at the end of the ' \
                  'simulation relative to the start. If spikes are still ' \
                  'observed at the end of the simulation, then it may be
that' \
                  ' you still have to fill the free energy profile (i.e. you
' \
                  'are exploring new regions of the phase space) and this
may' \
                  ' be related to the fact that your system free energy ' \
                  'profile is changing with time (due to an emerging ' \
                  'orthogonal degree of freedom).'

    desc_time = 'The free energy calculated by Metadynamics is approximated
' \
                'by the negative potential from the Metadynamics. This ' \
                'potential keeps increasing, and as a result, the estimates
' \
```

```
                'look like ever increasing wells. This is not an issue as we
' \
                'are only interested in the relative free energy difference
' \
                'between the points being sampled. In Metadynamics, ' \
                'those points not sampled have zero free energy, resulting '
\
                'in only those points with a negative free energy have their
' \
                'average value calculated.'

    desc_overlay = 'Since the only points that matter are those with a free
' \
                   'energy less than zero, it makes sense to average only '
\
                   'those points for which the free energy is within a ' \
                   'certain window from its lowest point and less then zero.
' \
                   'This corresponds to the points that are most sampled. '
\
                   'This plot contains the free energy aligned to this
common' \
                   ' region that is sampled. The points that are not sampled
' \
                   'are placed at a higher value 5 kcal/mol higher than all
' \
                   'the other points (an arbitrary offset, just to mark the
' \
                   'difference).'

    desc_err_with_time = 'This is the average distance between the aligned '
\
                         'free energies as a function of time. The time that
' \
                         'this converges provides you with a good estimate '
\
                         'for the transient time for your Metadynamics. This
' \
                         'should give the starting point for your averaging
' \
                         '(you might want to repeat the calculation after '
\
                         'you discover this value).'

    desc_prob_err_with_time = 'This is the average distance between the ' \
                              'probabilities of each block as a function of
' \
                              'time. In Well-Tempered Metadynamics one
should' \
                              ' reach a diffusive behaviour where at the end
' \
                              'of the simulation the entire domain is
equally' \
                              ' sampled in the CV space. This function
should' \
                              ' go to very low values together and the
extend' \
```

```
                              ' of the CV sampled should be maximum (i.e.
the' \
                              ' system should explore the CV space and not
be' \
                              ' stuck).'

    desc_avg = 'This is the average free energy surface. It is obtained by '
\
               'calculating the average value from the plot containing the'
\
               'overlays.'

    desc_1D_stdev = 'This is the distribution of the standard deviation of'
\
                    'free energy realization in the points where the average
' \
                    'has been calculated. The more it is shifted on the ' \
                    'right, the worse is the CV.'

    desc_2D_stdev = 'Standard deviation on the CV space'

    desc_block_probabilities = 'In Well-Tempered Metyadynamics, once the ' \
                               'hills are negligible you should make sure '
\
                               'that the system keep exploring the cv space
' \
                               'in a quasi-flat landscape.These below are '
\
                               'probabilities in each block (i.e. not from '
\
                               'the beginning of simulation) and they should
' \
                               'be almost identical at the end of the ' \
                               'simulation and they should show that the cv
' \
                               'space you want to describe is uniformly ' \
                               'sampled. If your system is stuck, then it is
' \
                               'likely you need to restart your simulation '
\
                               'with a higher kTemp.'


class SectionLine(platypus.Flowable):
    """
    Create a flowable line object
    """

    def __init__(self, width, height=0):
        platypus.Flowable.__init__(self)
        self.width = width
        self.height = height

    def __repr__(self):
        return f'Line(w={self.width}'

    def draw(self):
```

```
        """
        draw the line
        """
        self.canv.line(0, self.height, self.width, self.height)


class MetaDynamicsConvergenceAnalysis(PlotInfo):
    """
    Assess the convergence of the given MtD simulation
    """

    def __init__(self, meta_ana: meta.MetaDynamicsAnalysis,
                 cmd_args: argparse.Namespace):
        """
        :param meta_ana: MetaDynamicsAnalysis object
        :param cmd_args: Argument list
        """
        self.meta_ana = meta_ana
        self.cmd_args = cmd_args

        self.ktemp = None
        self.kbt = None
        self.free_energy_window = None
        self.steps = None
        self.last_kern = None
        self.kern_slice = None

        self.first_kern = 1
        self.max_val = -100000

        self.aligned_fes = {}
        self.common_region = {}
        self.probabilities_reshaped = {}

        self.temp_png_files = []
        self.free_energies = []
        self.projected_probabilities = []
        self.plottable_average_fes = []
        self.plottable_stdev = []

        self._preProcess()

        try:
            self._computeFESAverage()
        except ValueError as err:
            sys.exit(err)

        self._indexToGrid()

        # Re-run if we need to use probabilities to generate the offset
        if self.ktemp:
            try:
                self._computeFESAverage(histo=True)
            except ValueError as err:
                sys.exit(err)

        self._createAlignedFES()
```

```
        self._createPlottableData()

        pprint(self.average_fes)
        self._generateReport()

    def _preProcess(self) -> None:
        """
        Extract the simulation data
        """
        with open(self.cmd_args.cfg_file, 'r') as cfg_fh:
            cfg_sea = sea.Map(cfg_fh.read())

        metakeys = cfg_sea.ORIG_CFG.meta

        temperature = cfg_sea.integrator.temperature.T_ref.val

        if 'kTemp' in metakeys:
            self.ktemp = metakeys['kTemp'].val
            self.kbt = temperature * constants.GAS_CONSTANT

            # Rescale the hills to have the correct free energy
            self.meta_ana.original_height = numpy.copy(self.meta_ana.height)

            self.meta_ana.height = self.meta_ana.height * (
                self.ktemp + self.kbt) / self.ktemp

            # fes from bottom doesn't make much sense
            self.free_energy_window = 100000.0

            print('Simulation Type: Well-Tempered Metadynamics')
            print(f'kTemp: {self.ktemp}')
            print(f'kBT: {self.kbt} (kcal/mol)')
        else:
            print('Simulation Type: Classic Metadynamics')
            self.free_energy_window = self.cmd_args.free_energy_window

        print(f'Simulation Temp: {temperature}\n')

        self.num_kern, num_csv = self.meta_ana.centers.shape

        if self.cmd_args.integration_frequency:
            self.first_kern, self.last_kern, self.kern_slice =
self.cmd_args.integration_frequency

            # Determine the number of bins, round up in order to be able to
            # average the bin size if the given slice size is not divisible
            # by the number of kernels
            self.num_bins = math.ceil(
                (self.last_kern - (self.first_kern - 1)) / self.kern_slice)
        else:
            self.num_bins = self.cmd_args.bins
            self.last_kern = self.num_kern - 1

        if self.last_kern >= self.num_kern:
            raise ValueError(
                f'ERROR: \nThe last kernel specified ({self.last_kern}) is
not'
```

```
                f' less than the number of kernels ({self.num_kern}) in '
                f'{self.cmd_args.kerseq_file}')

        slice_width = numpy.linspace(self.first_kern - 1, self.last_kern,
                                     self.num_bins + 1)

        # Need to 0-index as self.steps is used to find data in lists
        self.steps = [(int(slice_width[i]), int(slice_width[i + 1]))
                      for i in range(self.num_bins)]

        if len(self.steps) < 1:
            raise ValueError('ERROR: Too few bins specified by the block
size')

        # Update the kern_slice value. The second value is taken in the
numpy
        # array as this is the first real value (first value is 0)
        self.kern_slice = int(slice_width[1])

    def _createHillDecayPlot(self) -> list[platypus.Table | SectionLine]:
        """
        For a Well-Tempered mtd simulation, plot the hill decay for the
given
        for the given range and frequency of hills

        :return: Return the flowable table containing png, description and
line
        """
        # Create a list of ints that reflect the first and last given hills
to
        # analyze, and the frequency
        hills = [self.steps[0][0]]
        hills.extend([lis[1] for lis in self.steps])

        # Reshape the energy heights to match the x-axis values
        energies = [self.meta_ana.original_height[hills]]

        png_file = 'heights_with_time.png'
        self.temp_png_files.append(png_file)

        self._createXYPlot(hills, energies, 'Number of hills',
                           'Free energy (kcal/mol)', png_file)

        return self._addPlot(png_file, self.height_header, self.desc_height)

    @staticmethod
    def _createXYPlot(x_data: list | numpy.ndarray,
                      y_data: list,
                      x_label: str,
                      y_label: str,
                      png_file: str,
                      err_y: list | None = None) -> None:
        """
        Create the xy line plot

        :param x_data: X-axis data
        :param y_data: Y-axis data
```

```
        :param x_label: X-axis label
        :param y_data: Y-axis label
        :param png_file: PNG file name
        :param err_y: Error bar data
        """
        mplchart.get_xy_plot(x_data,
                             *y_data,
                             err_y=err_y,
                             x_label=x_label,
                             y_label=y_label,
                             filename=png_file,
                             dpi=100,
                             size=(600, 400),
                             fontsize='medium')

    @staticmethod
    def _create2VarPlot(data: list,
                        png_file: str,
                        title: str | None = None) -> str:
        """
        Create a 2D contour plot

        :param data: Data to be plotted
        :param png_file: PNG file name
        :param title: Optional plot title

        :return: The filename the image of the plot was saved into (same
string
            as passed in with the filename keyword
        """
        return mplchart.get_2var_plot(data,
                                      x_label='CV1',
                                      y_label='CV2',
                                      filename=png_file,
                                      title=title,
                                      dpi=100,
                                      size=(400, 400),
                                      fontsize='x-small')

    def _createAlignedFES(self) -> None:
        """
        Align the free energy surface (FES). Even if the average was already
        calculated, recalculate on the subset of common points
        """
        for key, value in self.common_region.items():
            offset_vals = [dd + self.offset[j] for j, dd in
enumerate(value)]

            # In regular meta the convergence you canc calculate average and
std
            # welltempered meta is converging with time so it doesn't make
sense
            # to average or align
            if not self.ktemp:
                # Calculate the average FES and standard deviation
                avg = sum(offset_vals) / len(self.steps)
                stdev = statistics.pstdev(offset_vals)
```

```
                aligned = [avg, stdev]
                self.average_fes[key] = aligned

            self.aligned_fes[key] = offset_vals

        self.max_val = max(
            x for values in self.aligned_fes.values() for x in values)

    def _indexToGrid(self) -> None:
        """
        Create a reverse_map to go from index to grid
        """
        self.reverse_map = list(range(len(self.free_energies[0])))

        for fes in self.free_energies:
            ndim = len(fes[0]) - 1
            for i, line in enumerate(fes):
                mykey = ' '.join([str(line[a]) for a in range(ndim)])
                # Required to recast the values into the grid
                self.reverse_map[i] = mykey

    def _createPlottableData(self) -> None:
        """
        Create data for plotting
        """
        self.free_energies_aligned = copy.deepcopy(self.free_energies)

        ndim = len(self.free_energies[0][0]) - 1

        for n in range(len(self.steps)):
            for i, key in enumerate(self.reverse_map):
                if key in self.aligned_fes:
                    v = self.aligned_fes[key][n]
                else:
                    # Bogus number to undisplay what isn't in the common
region
                    v = self.max_val + 5

                self.free_energies_aligned[n][i][ndim] = v

                if n == 0 and not self.ktemp:
                    v1 = copy.deepcopy(self.free_energies[n][i])
                    v2 = copy.deepcopy(self.free_energies[n][i])

                    if key in self.aligned_fes:
                        v1[ndim] = self.average_fes[key][0]
                        v2[ndim] = self.average_fes[key][1]
                    else:
                        # Bogus number to undisplay what isn't in the common
region
                        v1[ndim] = self.max_val + 10
                        v2[ndim] = 0.0

                    self.plottable_average_fes.append(v1)
                    self.plottable_stdev.append(v2)

            if self.cmd_args.write_files:
```

```
                step_str = '_'.join(map(str, self.steps[n]))
                if self.ktemp:
                    out_fname = f'free_energy_{step_str}.fes'
                else:
                    out_fname = f'aligned_{step_str}.fes'

                self.meta_ana.writeFES(out_fname,
self.free_energies_aligned[n],
                                       self.meta_ana.bins, self.meta_ana.cv,
                                       'degrees')

        if self.cmd_args.write_files:
            if not self.ktemp:
                self.meta_ana.writeFES('stdev.fes', self.plottable_stdev,
                                       self.meta_ana.bins, self.meta_ana.cv,
                                       'degrees')

                self.meta_ana.writeFES('average.fes',
                                       self.plottable_average_fes,
                                       self.meta_ana.bins, self.meta_ana.cv,
                                       'degrees')

    def _computeFESAverage(self, histo: bool = False) -> None:
        """
        Calculate the average free energy surface (FES). If histo is True,
        calculate the probabilities

        :param histo: Calculate the probabilities
        """
        data = []
        if histo:
            print('Calculating Histograms')
        else:
            print('Calculating FES')

        for i, (start, end) in enumerate(self.steps):
            if histo:
                out_fname = f'{self.cmd_args.outfile}_{i}.prob'
                underlying_fes = None
            else:
                out_fname = f'{self.cmd_args.outfile}_{i}.fes'
                underlying_fes = data

            data.append(
                self._computeFESInInterval(start,
                                           end,
                                           out_fname=out_fname,
                                           histo=histo,
                                           underlying_fes=underlying_fes))

        common_region = self._averageThenAlign(data, histo=histo)

        if bool(self.average_fes) is False or bool(common_region) is False:
            raise ValueError('ERROR: Cannot average with no points')

        if histo:
            self.projected_probabilities = copy.deepcopy(data)
```

```
            self.probabilities_reshaped = copy.deepcopy(common_region)
        else:
            self.free_energies = copy.deepcopy(data)
            self.common_region = copy.deepcopy(common_region)

        self.offset = self._calculateOffsetsToReference(common_region)

    def _computeFESInInterval(self,
                              start: int,
                              end: int,
                              out_fname: str,
                              histo: bool = False,
                              underlying_fes=None) -> list:
        """
        Identify the grid from the ranges and the bins given in the cfg
file.
        For each gaussian, add it to the previous gaussian sum for each grid
        point. Adapted from meta.computeFES()

        :param start: Starting gaussian
        :param end: Ending gaussian
        :param histo: Calculate the probabilities
        :param out_fname: Output filename
        :param underlying_fes: Previous gaussians

        :return: List of lists where each nested list contains the free
energy
            data
        """
        # Set CV ranges
        self._setRanges()

        # Generate histogram edges
        shape, edges = self._generateEdges()

        # Reshape to get the required number of hills
        saved_centers, saved_scales, saved_height = self._reshape(start,
end)

        if histo:
            box = numpy.reciprocal(
                numpy.array([
                    (self.meta_ana.ranges[i][1] -
self.meta_ana.ranges[i][0]) /
                    (self.meta_ana.bins[i] - 1)
                    for i in range(len(self.meta_ana.bins))
                ]))

            self.meta_ana.height = numpy.full_like(self.meta_ana.height,
                                                   -0.0001)
            nh, ncol = self.meta_ana.scales.shape
            self.meta_ana.scales = numpy.repeat(box, nh).reshape(
                (ncol, nh)).transpose()

        self.free_energy_surface = numpy.zeros(shape)

        all_data = []
```

```
        step = 0
        for idx in numpy.ndindex(shape):
            all_data.append(self._getSurfaceData(idx, edges))
            step += 1

        if len(all_data[0]) - 1 != len(self.meta_ana.bins):
            raise ValueError(
                f'\nERROR: Bins provided ({self.meta_ana.bins}) does not '
                f'match the required dimensionality ({self.ndim})')

        if histo is True:
            # Normalize the data to one
            cum = 0.0
            for d in all_data:
                cum += d[len(d) - 1]
            for d in all_data:
                d[len(d) - 1] /= cum

        # If you have an underlying_fes in full dimensionality, sum it now
here
        if underlying_fes:
            for j in range(len(all_data)):
                all_data[j][self.ndim] += underlying_fes[-1][j][self.ndim]

        if self.cmd_args.write_files:
            self.meta_ana.writeFES(out_fname, all_data, self.meta_ana.bins,
                                   self.meta_ana.cv, 'degrees')

        # Reset to original data
        self.meta_ana.centers = numpy.copy(saved_centers)
        self.meta_ana.scales = numpy.copy(saved_scales)
        self.meta_ana.height = numpy.copy(saved_height)

        return all_data

    def _setRanges(self) -> None:
        """
        Set all CV ranges. Range for distance CVs is dynamically set
        """
        for i in range(len(self.meta_ana.ranges)):
            if not self.meta_ana.ranges[i]:
                self.meta_ana.ranges[i] = [
                    self.meta_ana.centers[:, i].min(),
                    self.meta_ana.centers[:, i].max()
                ]

    def _generateEdges(self) -> tuple[tuple[int], list[numpy.ndarray]]:
        """
        Generate the histogram bin edges for the FES calculation

        :return: Shape of the histogram, numpy array of the histogram bins
        """
        shape = tuple(self.meta_ana.bins)

        edges = []
        for i in range(len(self.meta_ana.bins)):
```

```
            bin_size = (self.meta_ana.ranges[i][1] -
self.meta_ana.ranges[i][0]
                       ) / (self.meta_ana.bins[i] - 1)

            edges.append(
                numpy.arange(self.meta_ana.ranges[i][0],
                             self.meta_ana.ranges[i][1] + bin_size,
bin_size))

        return shape, edges

    def _reshape(
            self, start: int,
            end: int) -> tuple[numpy.ndarray, numpy.ndarray, numpy.ndarray]:
        """
        Reshape the data to get the required number of hills

        :param start: First hill
        :param end: Last hill

        :return: Reshaped ndarrays
        """
        self.ndim = self.meta_ana.centers.shape[1]

        saved_centers = numpy.copy(self.meta_ana.centers)
        saved_scales = numpy.copy(self.meta_ana.scales)
        saved_height = numpy.copy(self.meta_ana.height)

        self.meta_ana.centers = numpy.resize(self.meta_ana.centers[start:],
                                             (end - start, self.ndim))

        self.meta_ana.scales = numpy.resize(self.meta_ana.scales[start:],
                                            (end - start, self.ndim))

        self.meta_ana.height = numpy.resize(self.meta_ana.height[start:],
                                            (end - start))

        return saved_centers, saved_scales, saved_height

    def _getSurfaceData(self, idx, edges: numpy.ndarray) -> list:
        """
        Collect the free energy surface data

        :param idx: Iteration counter
        :param edges: numpy array of the histogram bins

        return: List of all data
        """
        data = [edges[i][idx[i]] for i in range(len(idx))]

        self.free_energy_surface[idx] = self.meta_ana.evaluate(data)

        data.append(self.free_energy_surface[idx])

        angstrom_units = [
            'dist', 'rmsd', 'rmsd_alt', 'rmsd_symm', 'zdist', 'zdist0',
'rgyr',
```

```
            'rgyr_mass', 'whim1', 'whim2', 'whim3'
        ]

        # Convert the data values to degrees. This needs to happen after the
        # 'evaluate' call b/c evaluate expect the values to be in radians
        for i, cv in enumerate(self.meta_ana.cv):
            if cv in angstrom_units:
                continue
            else:
                data[i] = numpy.degrees(data[i])

        return data

    def _averageThenAlign(self,
                          free_energies: list,
                          histo: bool = False) -> dict[str, list[float]]:
        """
        Calculate the common region. Average over all the surfaces and align
all
        the FES to the average. Then calculate the common region and extract
it

        :param free_energies: List of free energies
        :param histo: Calculate the probabilities

        :return: Dictionary of free energy averages and a dictionary of
common
            surface points
        """
        common_region = defaultdict(list)
        for i, fes in enumerate(free_energies):
            for line in fes:
                mykey = ' '.join(map(str, line[:-1]))
                common_region[mykey].append(float(line[self.ndim]))

        # Calculate the average
        self.average_fes = {}
        self.average_fes2 = {}

        for key in common_region.keys():
            self.average_fes[key] = sum(common_region[key]) / float(
                len(common_region[key]))

        min_val = min(self.average_fes.values())

        if histo is False:
            for key in list(self.average_fes):
                value = self.average_fes[key]
                if value - min_val > self.free_energy_window or value > -
0.001:
                    self.average_fes.pop(key, None)
                    common_region.pop(key, None)

        return common_region

    def _calculateOffsetsToReference(self, common_region) -> numpy.zeros:
        """
```

```
        Calculate the surface offsets to the reference
x
        :param common_region: Dictionary of common surface points

        :return: Numpy zeros of energy offsets to the surface
        """
        x = numpy.zeros(len(common_region[list(common_region)[0]]))

        for i in range(len(x)):
            x[i] = numpy.mean([(self.average_fes[k] - common_region[k][i])
                               for k in self.average_fes])

        return x

    def _createTimePlot(self, data: dict, png_file: str,
                        header: platypus.Paragraph,
                        desc: str) -> list[platypus.Table | SectionLine]:
        """
        Create a plot of data over time

        :param data: Plottable data
        :param png_file: PNG file name
        :param header: Section header
        :param desc: Plot description

        :return: Return the flowable table containing png, description and
line
        """
        nkeys = len(data)
        errs = [0] * (len(self.steps) - 1)

        for i in range(1, len(self.steps)):
            for vals in data.values():
                errs[i - 1] += ((vals[i - 1] - vals[i])**2) / nkeys

        errs = [math.sqrt(j) for j in errs]
        hills = [lis[1] for lis in self.steps]

        # Remove first hill as we are binning values
        del hills[0]

        self._createXYPlot(hills, [errs], 'Number of hills',
                           'Free energy (kcal/mol)', png_file)

        return self._addPlot(png_file, header, desc)

    def _create1DPlot(self, x_data: list, y_data: list, png_file: str,
                      x_label: str, y_label: str, header:
platypus.Paragraph,
                      desc: str) -> list[platypus.Table | SectionLine]:
        """
        Create a 1D plot

        :param x_data: X-axis data
        :param y_data: Y-axis data
        :param png_file: PNG file name
        :param x_label: X-axis label
```

```
        :param y_data: Y-axis label
        :param header: Section header
        :param desc: Plot description

        :return: Return the flowable table containing png, description and
line
        """
        x_data = [f[0] for f in x_data[0]]
        y_data = [[v[self.ndim] for v in f] for f in y_data]

        self._createXYPlot(x_data, y_data, x_label, y_label, png_file)

        return self._addPlot(png_file, header, desc)

    def _create2DPlot(
            self,
            data: list,
            header: platypus.Paragraph,
            desc: str,
            aligned: bool = False,
            probability: bool = False
    ) -> list[platypus.Paragraph | platypus.Table]:
        """
        Create a 2D contour plot

        :param data: Plot data
        :param header: Section header
        :param desc: Plot description
        :param aligned: Data is aligned
        :param probability: Offset data are probabilities

        :return: Return the flowable table containing png, description and
line
        """
        plot_data = [header, platypus.Paragraph(desc, PARA_STYLE)]

        plots = []
        for i, free_energy in enumerate(data):
            if aligned:
                png_file =
f'{self.cmd_args.outfile}_aligned_{self.steps[i][1]}.png'
            elif probability:
                png_file =
f'{self.cmd_args.outfile}_probabilities_{self.steps[i][1]}.png'
            else:
                png_file = f'{self.cmd_args.outfile}_{self.steps[i][1]}.png'

            self.temp_png_files.append(png_file)

            title = f'{self.steps[i][1]} hills'

            plots.append(
                platypus.Image(
                    self._create2VarPlot(free_energy, png_file, title),
                    *SQUARE_IMG))

        for j in range(0, len(plots), 3):
```

```
            plot_data.append(platypus.Table([plots[j:j + 3]],
hAlign='LEFT'))

        return plot_data

    def _createPropAvg1DPlot(self) -> list[platypus.Paragraph |
platypus.Table]:
        """
        Plot the average probabilities

        :return: Return the flowable table containing png, description and
line
        """
        png_file = 'average_fes.png'
        self.temp_png_files.append(png_file)

        self._createXYPlot([v[0] for v in self.plottable_average_fes],
                           [[v[1] for v in self.plottable_average_fes]],
                           'CV Hills',
                           'Free energy (kcal/mol)',
                           png_file,
                           err_y=[[v[1] for v in self.plottable_stdev]])

        return self._addPlot(png_file, self.avg_header, self.desc_avg)

    def _createPropAvg2DPlot(self) -> list[platypus.Paragraph |
platypus.Table]:
        """
        Plot the average probabilities

        :return: Return the flowable table containing png, description and
line
        """
        png_file = 'average_fes.png'
        self.temp_png_files.append(png_file)

        self._create2VarPlot(self.plottable_average_fes, png_file)

        return self._addPlot(png_file,
                             self.avg_header,
                             self.desc_avg,
                             dimension=SQUARE_IMG)

    def _createSurfaceStd1DPlot(
            self) -> list[platypus.Paragraph | platypus.Table]:
        """
        Create a 1D plot of the standard deviations

        :return: Return the flowable table containing png, description and
line
        """
        [counts, free_energy] = numpy.histogram(
            [self.average_fes[key][1] for key in list(self.average_fes)])

        png_file = 'probability_stdev_freeenergy.png'
        self.temp_png_files.append(png_file)
```

```
        self._createXYPlot(free_energy[:-1], [counts], 'Free energy
(kcal/mol)',
                           'Counts', png_file)

        return self._addPlot(png_file, self.stdev_1D_header,
self.desc_1D_stdev)

    def _createSurfaceStd2DPlot(
            self) -> list[platypus.Paragraph | platypus.Table]:
        """
        Create a 2D contour plot of the standard deviations

        :return: Return the flowable table containing png, description and
line
        """
        png_file = 'stdev_freeenergy.png'
        self.temp_png_files.append(png_file)

        self._create2VarPlot(self.plottable_stdev, png_file)

        return self._addPlot(png_file,
                             self.stdev_2D_header,
                             self.desc_2D_stdev,
                             dimension=SQUARE_IMG)

    def _generateReport(self) -> None:
        """
        Create the final PDF report
        """
        output_pdf = f'{self.cmd_args.outfile}_report.pdf'
        self.doc = reportlab.platypus.SimpleDocTemplate(
            output_pdf, pagesize=reportlab.lib.pagesizes.A4)

        self.spacer = reportlab.platypus.Spacer(0, 0.1 * units.inch)

        self.temp_png_files = ['error_with_time.png']
        elements = []

        self._addParametersToReport(elements)

        elements.extend(
            self._createTimePlot(self.aligned_fes, 'error_with_time.png',
                                 self.err_with_time_header,
                                 self.desc_err_with_time))

        if self.ktemp:
            elements.extend(self._createHillDecayPlot())
            elements.append(platypus.PageBreak())

        if self.ndim == 1:
            elements.extend(
                self._create1DPlot(self.free_energies_aligned,
                                   self.free_energies, 'growing_fes.png',
                                   'CV Hills', 'Free energy (kcal/mol)',
                                   self.time_header, self.desc_time))

            if not self.ktemp:
```

```
                elements.append(platypus.PageBreak())
                elements.extend(
                    self._create1DPlot(self.free_energies_aligned,
                                       self.free_energies_aligned,
                                       'overlay_fes.png', 'CV Hills',
                                       'Free energy (kcal/mol)',
                                       self.overlay_header,
self.desc_overlay))

                elements.extend(self._createPropAvg1DPlot())
                elements.extend(self._createSurfaceStd1DPlot())

            else:
                elements.extend(
                    self._create1DPlot(self.projected_probabilities,
                                       self.projected_probabilities,
                                       'overlay_fes.png', 'CV',
'Probability',
                                       self.block_probabilities_header,
                                       self.desc_block_probabilities))

                elements.extend(
                    self._createTimePlot(self.probabilities_reshaped,
                                         'prob_error_with_time.png',
                                         self.prob_err_with_time_header,
                                         self.desc_prob_err_with_time))

        elif self.ndim == 2:

            if not self.ktemp:
                elements.extend(self._createSurfaceStd1DPlot())
            elements.append(platypus.PageBreak())

            elements.extend(
                self._create2DPlot(self.free_energies, self.time_header,
                                   self.desc_time))

            if not self.ktemp:
                elements.extend(
                    self._create2DPlot(self.free_energies_aligned,
                                       self.overlay_header,
                                       self.desc_overlay,
                                       aligned=True))

                elements.extend(self._createPropAvg2DPlot())
                elements.extend(self._createSurfaceStd2DPlot())
            else:
                self._create2DPlot(self.projected_probabilities,
                                   self.block_probabilities_header,
                                   self.desc_block_probabilities,
                                   probability=True)

        else:

            elements.append(
                platypus.Paragraph('Important Note', my_style['Heading2']))
```

```
            elements.append(
                platypus.Paragraph(
                    'Graphical output for free energies can '
                    'be produced only for one or two '
                    'dimensional free energies.', my_style['Bullet']))

            if not self.ktemp:
                elements.extend(self._createSurfaceStd1DPlot())
            else:
                elements.extend(
                    self._createTimePlot(self.probabilities_reshaped,
                                         'prob_error_with_time.png',
                                         self.prob_err_with_time_header,
                                         self.desc_prob_err_with_time))

        self.doc.build(elements)

        for png_file in self.temp_png_files:
            fileutils.force_remove(png_file)

        print(f'\nOutput written to {output_pdf}')

    def _addParametersToReport(self, elements) -> None:
        """
        Add the standard header to the PDF output file
        """
        elements.append(
            platypus.Paragraph('Metadynamics Convergence Analysis',
                               my_style['Title']))

        elements.append(
            platypus.Paragraph('Analysis Parameters', my_style['Heading2']))
        elements.append(
            platypus.Paragraph(f'<b>Starting Hill:</b> {self.first_kern}',
                               my_style['Bullet']))
        elements.append(
            platypus.Paragraph(f'<b>Final Hill:</b> {self.last_kern}',
                               my_style['Bullet']))
        elements.append(
            platypus.Paragraph(
                f'<b>Interval between Hills:</b> {self.kern_slice}',
                my_style['Bullet']))
        elements.append(
            platypus.Paragraph(f'<b>Number of Blocks:</b>
{len(self.steps)}',
                               my_style['Bullet']))

        if self.ktemp:
            ktemp_str = platypus.Paragraph(
                f'<b>Well-Tempered Acceleration Factor (kTemp):</b>
{self.ktemp} kcal/mol',
                my_style['Bullet'])
        else:
            ktemp_str = platypus.Paragraph(
                f'<b>Free Energy Window used for Averaging (kTemp):</b>
{self.free_energy_window} kcal/mol',
                my_style['Bullet'])
```

```
        elements.append(ktemp_str)
        elements.append(self.spacer)
        elements.append(SectionLine(self.doc.width))

    def _addPlot(
        self,
        png_file: str,
        header: platypus.Paragraph,
        desc: str,
        dimension: tuple = RECTANGLE_IMG
    ) -> list[platypus.Paragraph | platypus.Table]:
        """
        Create the flowable tabel with the plot data and text

        :param png_file: PNG file name
        :param header: Section header text
        :param desc: Plot description
        :param dimension:

        :return: Return the flowable table containing png, description and
line
        """
        flow_data = [header]

        tbl = platypus.Table([[
            platypus.Image(png_file, *dimension),
            platypus.Paragraph(desc, PARA_STYLE)
        ]],
                             style=TABLE_STYLE)

        flow_data.append(tbl)
        flow_data.append(SectionLine(self.doc.width))

        return flow_data


def parse_args(argv: tuple[str]) -> argparse.Namespace:
    """
    Parse the command line options.

    :param argv: Argument list

    :return: All script arguments and options
    """
    parser = cmdline.create_argument_parser(
        description=__doc__,
        formatter_class=argparse.RawDescriptionHelpFormatter)

    parser.add_argument('cfg_file',
                        type=parserutils.type_file,
                        help='Metadynamics cfg file')

    parser.add_argument('kerseq_file',
                        type=parserutils.type_file,
                        help='Metadynamics kerseq file')
```

```
    parser.add_argument('outfile', help='Output file base name')

    group = parser.add_mutually_exclusive_group(required=True)

    group.add_argument(
        '-integration_frequency',
        metavar='\b',
        type=int,
        nargs=3,
        help='Specify the frequency of integrating the hills in order to '
        'generate the number of bins. Three values are required, the first '
        'hill, the last hill and the size of the block to analyze. The size
of'
        ' the block must results in at least two bins being analyzed.')

    group.add_argument(
        '-bins',
        metavar='\b',
        type=int,
        help='Specify the number of bins to analyze. The minimum number of '
        'bins to analyze is 2')

    parser.add_argument(
        '-free_energy_window',
        metavar='\b',
        type=float,
        default=2.0,
        help='Free energy window from the minima used to estimate the
average. '
        'Default: 2.0')

    parser.add_argument('-write_files',
                        action='store_true',
                        help='Write out .fes and .prob files')

    args = parser.parse_args(argv)

    if args.bins and args.bins < 2:
        parser.error('More than 1 bin needs to be specified with -bins')

    return args


def main(*argv: str) -> None:
    """
    Main body of the script
    """
    print('Launching Metadynamics Convergence Analysis Script\n')

    cmd_args = parse_args(argv)

    try:
        meta_ana = meta.MetaDynamicsAnalysis(cmd_args.kerseq_file,
                                             inp_fname=cmd_args.cfg_file)
    except AttributeError:
        sys.exit(f'Invalid Metadynamics cfg file: {cmd_args.cfg_file}')
```

```
    try:
        MetaDynamicsConvergenceAnalysis(meta_ana, cmd_args)
    except ValueError as err:
        print(err)
        sys.exit(1)


if __name__ == '__main__':
    main(*sys.argv[1:])
```
