# Supplementary material for: 3D variability analysis reveals a hidden conformational change controlling ammonia transport in human asparagine synthetase
Source: Nat Commun. 2024 Dec 3;15:10538. doi: 10.1038/s41467-024-54912-9 (PMC11615228; doi:10.1038/s41467-024-54912-9)
Supplement: Supplementary file 5 — Supplementary Data 3 [file 41467_2024_54912_MOESM5_ESM.pdf]

Reliability and reproducibility checklist for molecular dynamics simulations<br>*All boxes must be marked YES by acceptance unless an N/A option is available	Yes	N/A	Response<br>(Please state where this information can be found in the text)
**1. Convergence of simulations and analysis**			
1a. Is an evaluation presented in the text to show that the property being measured has equilibrated in the simulations (*e.g.* time-course analysis)?	☒		Supplementary Figures
1b. Then, is it described in the text how simulations are split into equilibration and production runs and how much data were analyzed from production runs?	☒		Methods
1c. Are there at least 3 simulations per simulation condition with statistical analysis?	☒		Supplementary Figures 18, 24 and 33
1d. Is evidence provided in the text that the simulation results presented are independent of initial configuration?	☒		Supplementary Figures 18, 24 and 33
**2. Connection to experiments**			
2a. Are calculations provided that can connect to experiments (*e.g.* loss or gain in function from mutagenesis, binding assays, NMR chemical shifts, J-couplings, SAXS curves, interaction distances or FRET distances, structure factors, diffusion coefficients, bulk modulus and other mechanical properties, *etc.*)?	☒		Results
**3. Method choice**			
3a. Is it described in the text what force field and water model are used and why?	☒		Methods
3b. Do simulations contain membranes, membrane proteins, intrinsically disordered proteins, glycans, nucleic acids, polymers, or cryptic ligand binding?	☐	☒	Response not needed if **N/A**
If 3b is **YES**, are enhanced sampling methods used?	☒	☐	Enhanced sampling is used to obtain potential energy surfaces for the R142 side chain in the presence and absence of the aspartyl-AMP ligand.
If enhanced sampling methods are used, are the convergence criteria clearly stated?	☒		Convergence data are shown in Supplementary Figures 35 and 36. A script for analyzing the enhanced sampling data (uploaded into the Zenodo repository) is provided as Supplementary Data 2
If 3b is **YES**, is it explained in the text why or why not enhanced sampling methods are used?	☒		Results and Supplementary Fig. 19
**4. Code and reproducibility**			
4a. Is a table provided describing the system setup, such as simulation box dimensions, total number of atoms, total number of water molecules, salt concentration, lipid composition (number of molecules and type)?	☒		Source data
4b. Is it described in the text what simulation and analysis software and which versions are used?	☒		A list of the software packages used to perform

			the MD simulations, and to make Supplementary Movies 11-14, is provided in the Reporting Summary
4c. Are initial coordinate and simulation input files and a coordinate file of the final output provided as supplementary files or in a public repository?	☒		All MD coordinate and trajectory files are available the Zenodo repository, as shown in Supplementry Table 8.
4d. Is there custom code or custom force field parameters?	☒	☐	Custom parameters were used for the β-aspartyl-AMP ligand
If **YES**, are they provided as supplementary profiles or in a public repository?	☒		These custom parameters are provided as Supplementary Data 1
