# Supplementary material for: 3D variability analysis reveals a hidden conformational change controlling ammonia transport in human asparagine synthetase
Source: Nat Commun. 2024 Dec 3;15:10538. doi: 10.1038/s41467-024-54912-9 (PMC11615228; doi:10.1038/s41467-024-54912-9)
Supplement: Supplementary file 6 — Reporting Summary [file 41467_2024_54912_MOESM6_ESM.pdf]

nature portfolio

Corresponding author(s): Stefano Alcaro, Wen Zhu, Yuro Takagi, Nigel Richards

Last updated by author(s): Nov 4, 2024

Reporting Summary

Nature Portfolio wishes to improve the reproducibility of the work that we publish. This form provides structure for consistency and transparency in reporting. For further information on Nature Portfolio policies, see our Editorial Policies and the Editorial Policy Checklist.

Statistics

For all statistical analyses, confirm that the following items are present in the figure legend, table legend, main text, or Methods section.

n/a	Confirmed	
☐	☒	The exact sample size (*n*) for each experimental group/condition, given as a discrete number and unit of measurement
☒	☐	A statement on whether measurements were taken from distinct samples or whether the same sample was measured repeatedly
☒	☐	The statistical test(s) used AND whether they are one- or two-sided<br>*Only common tests should be described solely by name; describe more complex techniques in the Methods section.*
☒	☐	A description of all covariates tested
☒	☐	A description of any assumptions or corrections, such as tests of normality and adjustment for multiple comparisons
☐	☒	A full description of the statistical parameters including central tendency (e.g. means) or other basic estimates (e.g. regression coefficient) AND variation (e.g. standard deviation) or associated estimates of uncertainty (e.g. confidence intervals)
☒	☐	For null hypothesis testing, the test statistic (e.g. *F*, *t*, *r*) with confidence intervals, effect sizes, degrees of freedom and *P* value noted<br>*Give P values as exact values whenever suitable.*
☒	☐	For Bayesian analysis, information on the choice of priors and Markov chain Monte Carlo settings
☒	☐	For hierarchical and complex designs, identification of the appropriate level for tests and full reporting of outcomes
☒	☐	Estimates of effect sizes (e.g. Cohen's *d*, Pearson's *r*), indicating how they were calculated

Our web collection on statistics for biologists contains articles on many of the points above.

Software and code

Policy information about availability of computer code

Data collection	All software used in this paper is commercially or freely available. The software used to collect cryo-EM data was EPU (FEI/ThermoFisher).
Data analysis	For the structural studies, data were processed using cryoSPARC v3.2.2 and v4.4.1, PHENIX (the PHENIX.varref analysis tools are already embedded in the current release) and Coot. EM structures were visualised in UCSF Chimera and ChimeraX. MD and metadynamics simulations employed Maestro and DESMOND v5.3 (Schrodinger Suite 2018-01). Simulation data was analysed using CPPTRAJ and the CAVER 3.0 plugin for PyMOL. Supplementary Movies 11-14 were made with VMD v1.9.4a55. The script used to evaluate convergence of the WT-MTD calculations has been uploaded as Supplementary Data 2.

For manuscripts utilizing custom algorithms or software that are central to the research but not yet described in published literature, software must be made available to editors and reviewers. We strongly encourage code deposition in a community repository (e.g. GitHub). See the Nature Portfolio guidelines for submitting code & software for further information.

Data

Policy information about availability of data

All manuscripts must include a data availability statement. This statement should provide the following information, where applicable:

- Accession codes, unique identifiers, or web links for publicly available datasets
- A description of any restrictions on data availability
- For clinical datasets or third party data, please ensure that the statement adheres to our policy

Structural data have been deposited in the PDB (http://doi.org/10.2210/pdb8SUE/pdb and http://doi.org/10.2210/pdb9B6G/pdb) and the Electron Microscopy Data Bank (https://www.ebi.ac.uk/emdb/EMD-40764 and https://www.ebi.ac.uk/emdb/EMD-44253). Supplementary movies 1-14, EM map/model data, 3DVA-derived data, including, coordinate files for the 100 sampled structures, and all MD and metadynamics trajectories are available in the Zenodo repository (https://zenodo.org). DOI information is provided in the Data Availability statement and Supplementary Table 6.

Research involving human participants, their data, or biological material

Policy information about studies with human participants or human data. See also policy information about sex, gender (identity/presentation), and sexual orientation and race, ethnicity and racism.

Reporting on sex and gender	N/A
Reporting on race, ethnicity, or other socially relevant groupings	N/A
Population characteristics	N/A
Recruitment	N/A
Ethics oversight	N/A

Note that full information on the approval of the study protocol must also be provided in the manuscript.

Field-specific reporting

Please select the one below that is the best fit for your research. If you are not sure, read the appropriate sections before making your selection.

☒ Life sciences ☐ Behavioural & social sciences ☐ Ecological, evolutionary & environmental sciences

For a reference copy of the document with all sections, see nature.com/documents/nr-reporting-summary-flat.pdf

Life sciences study design

All studies must disclose on these points even when the disclosure is negative.

Sample size	The number of replicate kinetic measurements and MD simulations were chosen to conform with standard literature protocols.
Data exclusions	No data were excluded.
Replication	Kinetic assays and MD simulationss were replicated as described in the text.
Randomization	Our study did not require any sample randomization.
Blinding	Our study did not require any blinding.

Reporting for specific materials, systems and methods

We require information from authors about some types of materials, experimental systems and methods used in many studies. Here, indicate whether each material, system or method listed is relevant to your study. If you are not sure if a list item applies to your research, read the appropriate section before selecting a response.

Materials & experimental systems

n/a	Involved in the study	
☒	☐	Antibodies
☐	☒	Eukaryotic cell lines
☒	☐	Palaeontology and archaeology
☒	☐	Animals and other organisms
☒	☐	Clinical data
☒	☐	Dual use research of concern
☒	☐	Plants

Methods

n/a	Involved in the study	
☒	☐	ChIP-seq
☒	☐	Flow cytometry
☒	☐	MRI-based neuroimaging

Eukaryotic cell lines

Policy information about cell lines and Sex and Gender in Research

Cell line source(s)	Spodoptera frugiperda (Sf9) cells were purchased from Expression Systems, Inc (Catalog #94-001S)
Authentication	Cell lines were purchased from a commercial supplier and were not further authenticated.
Mycoplasma contamination	The cell line used to express WT and R142I ASNS samples tested negative for mycoplasma contamination.
Commonly misidentified lines (See ICLAC register)	No commonly misidentified cell lines were used in this study.
